# Silicon and the Plant Extracellular Matrix

**DOI:** 10.3389/fpls.2016.00463

**Published:** 2016-04-12

**Authors:** Gea Guerriero, Jean-Francois Hausman, Sylvain Legay

**Affiliations:** Environmental Research and Innovation Department, Luxembourg Institute of Science and TechnologyEsch-sur-Alzette, Luxembourg

**Keywords:** silicic acid, biosilicification, cell wall, priming, metabolism

## Abstract

Silicon (Si) is one of the most abundant elements on earth. Although not considered essential for the growth and development of higher plants, it is nonetheless known to increase vigor and to play protective roles. Its protective effects include for instance alleviation of (a)biotic stress damages and heavy metal toxicity. Si was shown to interact with several components of the plant cell walls in the form of silica (SiO_2_). In plants SiO_2_ promotes strengthening of the cell walls and provides increased mechanical support to the aerial parts. The relationship SiO_2_-plant cell wall has been well documented in monocots and pteridophytes, which are known Si accumulators, while much less is known on the interaction of Si with the cell walls of dicots. We here provide a concise up-to-date survey on the interaction between Si and plant cell wall components by focussing on cellulose, hemicelluloses, callose, pectins, lignin, and proteins. We also describe the effects of Si on cell wall-related processes by discussing the published results in both monocots and dicots. We conclude our survey with a description of the possible mechanisms by which Si exerts priming in plants.

## Introduction

Silicon (Si) occurs abundantly in the earth’s crust and it is considered a beneficial element for plants. Si-deprived plants are structurally weaker, more susceptible to infections and abiotic stresses (Epstein, 1999). Si also enhances organogenesis and embryogenesis in *in vitro*-grown plants (Sivanesan and Park, 2014).

The form of Si that is taken up by plants in soil waters is silicic acid, Si(OH)_4_ (Exley, 1998, 2015), a very weak acid (pka >9.5). The entry of Si(OH)_4_ into the roots most likely takes place following water (Exley, 2015 and references therein), via either an apoplastic or symplastic route. The symplastic route requires the presence of water channels (aquaporins belonging to the Nod26-like intrinsic proteins, NIPs); NIPs permeable to Si(OH)_4_ have been identified in different plants, e.g., *Equisetum arvense*, monocots and dicots (Ma et al., 2006; Chiba et al., 2009; Mitani et al., 2009a,b; Grégoire et al., 2012; Deshmukh et al., 2013). The selective permeability of aquaporins, which determines the resistance to Si(OH)_4_ movement, is responsible for its concentration within specific plant comparments (Exley, 2015). It was recently shown that a specific spacing of 108 amino acids between the NPA domains of NIP aquaporins determines Si permeability (Deshmukh et al., 2015).

**FIGURE 1:**
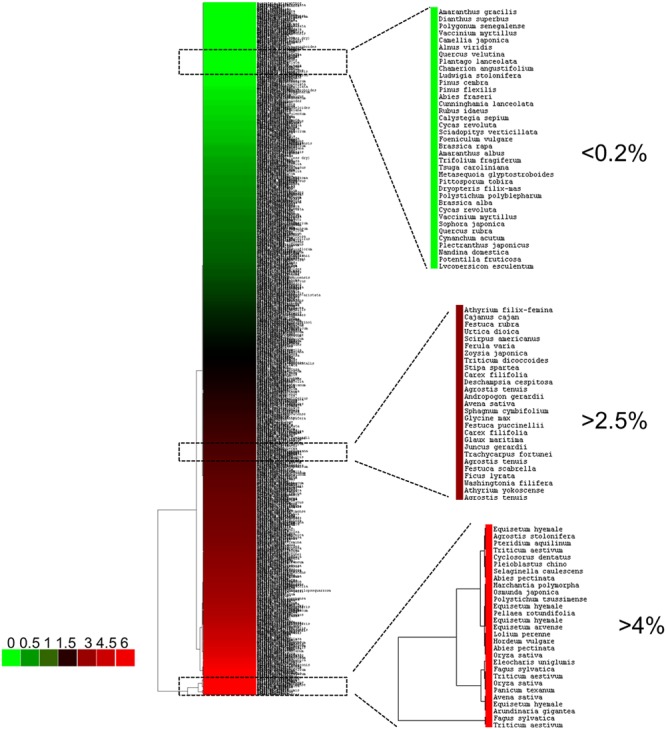
**Hierarchical clustering of representative land plants based on their SiO_2_ content.** The data used correspond to those published by Trembath-Reichert et al., 2015 (Dataset S1 of this publication). The hierarchical clustering (Euclidean distance, complete linkage) was performed with Cluster 3.0 (Eisen et al., 1998) and visualized with Java TreeView (Saldanha, 2004). The colorbar indicates the SiO_2_ content (% wt). The boxed regions of the tree correspond to representatives belonging to different Si-classes.

Plants are typically classified into three main categories, i.e., Si-excluders, Si-intermediate types and Si-accumulators (Mitani and Ma, 2005) (**Figure 1**). To the first group belong plants of the order Equisetales, Poales, and Cyperales, which accumulate >4% Si (of shoot dry weight) in their tissues (Currie and Perry, 2007); plants showing >1% Si (between 2 and 4%, e.g., Cucurbitales and Urticales) are of the intermediate type; the Si-excluders show <0.5% Si (Epstein, 1994; Ma et al., 2007).

Si exerts positive effects on plants subjected to exogenous stresses, while the addition of Si to unstressed plants results in fewer changes, e.g., in gene expression (Fauteux et al., 2006; Detmann et al., 2013). Nevertheless, a study on unstressed rice plants showed that Si improves crop yield via an alteration of the source-sink relationship, triggered by the remobilization of amino acids to sustain the increased nitrogen requirement of the grains (Detmann et al., 2012, 2013). Si may therefore behave as a signaling factor and exerts its positive effects by redirecting the primary metabolism of plants.

Si protects salt-stressed wheat seedlings by decreasing the concentration of Na^+^ (Azeem et al., 2015). Si likewise protects cucumber against ion toxicity caused by salt stress by improving the root hydraulic conductance and via an increase in polyamines (which block non-selective cation channels and inhibit the influx of Na^+^) (Wang et al., 2015).

Many thorough reviews have been published on the phytoprotective role of Si (e.g., Van Bockhaven et al., 2013; Adrees et al., 2015; Rizwan et al., 2015) and on the mechanisms of biosilicification in plants (Bauer et al., 2011; Exley, 2015), while no review has so far concentrated on the plant cell wall/Si binomium. In this minireview we fill this gap by focussing on the relationship Si/plant cell wall. More specifically we: (1) discuss the role of some cell wall components (polysaccharides and lignin) and proteins in templating SiO_2_ deposition (2) describe the direct effects of Si on plant cell walls, (3) illustrate the role of Si on cell wall-related processes, (4) describe the effects of Si on plant metabolism and (5) discuss the ways by which Si might exert its priming role in plants.

### Biosilicification and Plant Cell Wall Components

Some reports linking SiO_2_ deposition with cell wall polysaccharides are available (*vide infra*), however, the molecular details explaining the cell wall polysaccharide-assisted biosilicification in plants are only fragmentary. The role of cell wall macromolecules (both polysaccharides and lignin) in templating biosilicification might be an entirely passive mechanism. The function of cell wall components might be that of sequestering otherwise cytotoxic silica particles formed by Si(OH)_4_ autocondensation: as recently discussed by Exley (2015), the two conditions to avoid cytotoxicity of silicification are (1) controlling the size of silica nanoparticles so that they remain below 5 nm and (2) the involvement of cellular components resistant to denaturation in the case of nanoparticles above 5 nm. Cell wall polysaccharides (as for instance the non-cellulosic ones, like callose, *vide infra*) might “soak up” small particles like a “sponge” (Exley, 2015) and entrap them in a network hindering their growth to sizes above 5 nm. Other more recalcitrant components, like the macromolecule lignin, might be involved in sequestering larger particles.

A representative example of the intimate relationship between Si and cell wall polysaccharides comes from the Si-accumulator rice. Commelinoid monocots (e.g., cereals, like rice) possess “type II” cell walls which are characterized by less pectins and more phenylpropanoids compared to “type I” dicot cell walls. Type II cell walls contain also glucuronoarabinoxylan cross-linking cellulose and mixed-linkage glucans (Yokoyama and Nishitani, 2004). In “type II” cell walls Si might have a similar structural role as boron in dicots, where it cross-links pectic polysaccharides and thereby maintains the integrity (He et al., 2013 and references therein). It is plausible that the complexation of Si with cell wall macromolecules takes place via the stabilization of sugars, in a manner analogous to the borate-mediated formose reaction (He et al., 2013 and references therein).

In this section of the review we will discuss about the literature data linking biosilicification with cellulosic and non-cellulosic polysaccharides, lignin, and proteins.

Cellulose is the chief load-bearing component of plant cell walls and it was speculated that since it forms tightly packed crystalline microfibrils, there would be little chance to bind Si (He et al., 2015). However, Perry and Lu (1992) reported that the addition of insoluble cellulose to an aqueous solution of octahedral silicon complex favored the formation of ordered aggregates (with the shape of sheets) at neutral pH (pH 6.67–7.1), while in the absence of cellulose, disordered aggregates of varying diameters were observed. This result suggests a potential role of cellulose in controlling ordered SiO_2_ deposition.

Callose is a component of plant cell walls which is synthesized by membrane bound glycosyltransferases of family 48 (callose synthases) and plays an important role in both plant development and stress response (Chen and Kim, 2009). The first proof of a link between silicification and callose was shown in *Equisetum* (Law and Exley, 2011), a vascular plant accumulating high levels of SiO_2_. Using the fluor PDMPO and fluorescence microscopy, the authors observed that the deposition of SiO_2_ followed the deposition of callose and this was particularly evident in the horsetail stomata. Interestingly, callose was able to induce the precipitation of SiO_2_ *in vitro* when undersaturated solutions of Si(OH)_4_ were used. The authors speculate that the hydroxyl groups of the glucose units in the callose homopolymer might be involved in initiating the first steps of autocondensation, by contributing to overcoming the energy barrier required for the autocondensation reaction.

Horsetail cell walls are also characterized by the presence of mixed-linkage glucans (Sørensen et al., 2008), a feature shared with the phylogenetically distant order Poales. It was suggested that mixed-linkage glucans act as template for silicification (Fry et al., 2008), an observation recently corroborated in rice by Kido et al. (2015). The overexpression of the (1;3,1;4)-β-D-glucanase *OsEGL1* in rice did not affect the total concentration of Si, but affected the distribution profile of Si and the mechanical properties of the leaf blades after supplementation of Si (Kido et al., 2015). In rice mixed-linkage glucans can control, together with other macromolecules, the polymerization sites and/or the total accumulation of Si (Kido et al., 2015).

A study on rice cell suspension cultures carried out using inductively coupled plasma mass spectrometry and X-ray photoelectron spectroscopy showed that Si occurs in the cell wall, where it is firmly associated with wall macromolecules (mainly hemicellulose components, via Si-O-C bonds; He et al., 2015). Si additionally improves both the regeneration of cell walls in protoplasts (He et al., 2015) and the mechanical properties of rice cell walls. It increases the length of cellulose filaments and makes cellulose microfibrils denser (He et al., 2015). The association of Si with hemicelluloses is important for the *in vivo* Cd detoxification in rice cell suspension culture: when the concentration of Cd^2+^ increased beyond the natural binding ability of rice cell walls, Si-supplemented cells showed a smaller net influx of the toxic ion in the cells (Ma et al., 2015).

A functional correlation between Si and mixed-linkage glucans was established in rice (Kido et al., 2015).

In the fern *Adiantum raddianum* pectic homogalacturonan epitopes were found in the SiO_2_-enriched cell walls (Leroux et al., 2013). In particular, the LM6 antibody recognizing pectic arabinan, showed labeling of the silicified outer cell walls of the fiber-like epidermal cells (Leroux et al., 2013). The authors hypothesize that the silicified outer cell walls of these cells support the leaf lamina during expansion before the start of secondary cell wall formation and suggest a role for pectic arabinan in SiO_2_ deposition. A further result linking pectin and SiO_2_ deposition comes from a study carried out on *Equisetum hyemale*, where a colocalization of pectin and SiO_2_ was observed (Gierlinger et al., 2008).

Some studies in the literature have shown the association of Si with lignin. Inanaga et al. (1995) showed that in rice cell walls SiO_2_ interacts with the aromatic ring or phenolic acid of the lignocarbohydrate complex. Later, by using transmission electron microscopy coupled with electron energy loss microanalysis, Watteau and Villemin (2001) confirmed this association in another species, by noticing that in beech root cell walls SiO_2_ was associated with polyphenolic compounds.

In *Cucurbita* fruits the formation of phytoliths and lignification are determined by the same dominant genetic locus, *Hr* (hard rind) (Piperno et al., 2002), a finding which illustrates the mechanical role of SiO_2_ and confirms the existing link between lignin and SiO_2_ deposition.

A study carried out on Si and lignin from rice showed that Si alone could not precipitate in borax aqueous solution (pH 10.05), while macrolignin (and not the lignin residue) could trigger SiO_2_ deposition via the formation of 5–6 coordinated bonds with Si (Fang and Ma, 2006).

More recently, lignification of rice silica cells was shown to precede Si deposition in their lumen (Zhang et al., 2013).

### Biosilicification and Proteins

Besides cell wall polysaccharides/macromolecules, *in vitro* studies have shown that the specific amino acid composition of cell wall-localized proteins affects biosilicification (Currie and Perry, 2007 and references therein). Amino acids with positively charged side-chains can associate with the negatively charged silica species via electrostatic interactions, thereby favoring biosilicification. In this respect, it is noteworthy to mention the study on *Cucumis sativus*, in which a gene encoding a cell wall proline-rich protein (*PRP1*), that contains C-terminal repetitive sequences with clusters of positively charged amino acids (namely Arg and Lys), was shown to be specifically induced in systemically resistant plants (Kauss et al., 2003). Interestingly, synthetic peptides corresponding to the repetitive sequences of PRP1 induced the polymerization of Si(OH)_4_ to SiO_2_, a result suggesting that the protein induces cell wall strengthening in cucumber via SiO_2_ deposition, upon induction of systemic acquired resistance (Kauss et al., 2003). The formation of SiO_2_ thus provides a “shield” at the sites of fungal penetration.

We also mention here the role of a serine-rich protein from mangrove (*Rhizophora apiculata*), which, although not predicted to be localized in the cell wall, was shown to play a positive role in Si accumulation (Sahebi et al., 2015). The gene coding for this protein was shown to be upregulated in the roots of Si-treated mangrove (Sahebi et al., 2014) and to induce Si accumulation in both leaves and roots when expressed in a heterologous host, namely *Arabidopsis thaliana* (Sahebi et al., 2015). It is interesting to note here that in diatoms, which are well-known biosilicifying organisms, proteins rich in hydroxyamino acids are overrepresented in cell wall hydrolysates and may create ester bonds with Si(OH)_4_ (Kröger and Poulsen, 2008).

### Direct Effects of Si on Plant Cell Wall

Si alleviates the toxic effects associated with heavy metals in both monocots and dicots (Parrotta et al., 2015 and references therein). For example, in maize plants Si favors the binding of Cd to the apoplasmic fraction of the shoots, while in the roots it stimulates the formation of suberin lamellae and the maturation of vascular tissues (Vaculík et al., 2012); Si also favors the binding of Al to the apoplast of maize root apex (Wang et al., 2004). In wheat seedlings Si deposition near the endodermis decreases the porosity of the Casparian strip and reduces the apoplasmic transport of Cd (Rizwan et al., 2016). In rice cell suspension culture, Si accumulates in the cell wall under the form of an organosilicon compound which binds Cd thereby inhibiting its uptake (Liu et al., 2013).

In cucumber and tomato Si alleviates the effects of Cd toxicity via distinct mechanisms involving, among other factors, organic acid levels and cell wall polysaccharides (Wu et al., 2015). This finding shows species-specific effects of Si: while in tomato the root-to-shoot translocation is decreased, in cucumber roots the uptake of Cd is reduced.

Si also induces modifications in the cell wall mechanical properties and composition, notably in the roots of plants (e.g., Hattori et al., 2003; Vaculík et al., 2012; Rizwan et al., 2016) and amorphous SiO_2_ deposits are found in association with plant cell walls, where the cell wall macromolecules template biosilicification (e.g., Fry et al., 2008; Law and Exley, 2011). Therefore, the benefits of Si in stressed plants are (partly) due to its effects on cell wall biosynthesis and its mechanical role. SiO_2_ acts as herbivore deterrent (Farooq and Dietz, 2015), indeed SiO_2_-rich lignocellulosic biomass is appreciated in construction as the material is more durable (e.g., hemp woody core; Guerriero et al., 2016).

Another study on the cell wall effects of Si supplementation in Si-accumulators (rice and maize) and non-accumulators (onion) showed an increased formation of Casparian bands in the exodermis. The Casparian band development was, however, not accompanied by an increase in lignin nor suberin (esterified phenolic compounds, which determine the aromatic part of suberin, decreased after depolymerization in rice and maize roots; Fleck et al., 2015). The authors discussed that the increased formation of Casparian bands was due to the interaction of Si with phenolic compounds.

### Effects of Si on Cell Wall-Related Processes

The addition of Si influences cell wall-related processes in both monocots and dicots. Understanding more about the relationship Si-cell wall processes can help devise biotechnological strategies aimed at boosting the biosynthesis of lignocellulosic biomass, an important renewable commodity for mankind (Guerriero et al., 2014). In this paragraph we illustrate the effects of Si nutrition on the cell walls of monocots (by taking rice as model) and dicots. We also describe the effects on Si deprivation on the cell walls of rice, in the light of its impact on secondary cell wall biosynthesis.

The addition of Si to rice plants induces a decrease in radial oxygen loss in the basal part of the adventitious roots (Fleck et al., 2011). This phenomenon is due to lignified sclerenchyma cells and suberized exodermis and endodermis (Fleck et al., 2011). The authors indeed observed that suberization of Si-supplied roots started already at 4–5 cm from the root tip, while in control plants this process started at 8–9 cm from the tip; additionally, suberization in the endodermis of Si-supplied roots was observed already at 1–2 cm, while no suberin was detected in control roots. The increased lignification of Si-supplemented roots was due to an increase in the transcript abundance of genes involved in monolignol biosynthesis, namely phenylalanine ammonia lyase and 4-coumarate:CoA ligase, and monolignol transport/polymerization, i.e., ABC transporters and peroxidases (Fleck et al., 2011). The increased expression of ABC transporters and peroxidases is known to be also associated with an increased suberin biosynthesis (Legay et al., 2015).

Si affects potato tuber skin by increasing suberization and by significantly upregulating suberin-associated 3-ketoacyl-CoA synthase (which is involved in suberin deposition). Si was also shown to decrease the age-dependent changes in the skin cell area, via a structural stabilization of the cell wall, thereby delaying senescence of the tuber skin (Vulavala et al., 2015).

Si deprivation in rice induces cell wall thickening to compensate for the lack of SiO_2_ and hence the decreased mechanical strength. An increase in secondary cell wall biosynthesis (via the upregulation of lignin-related genes and secondary cell wall cellulose synthases) was indeed observed (Yamamoto et al., 2012). In particular, with respect to Si-supplementation, an augmented cellulose deposition was observed in short cells of Si-deprived plants, which normally accumulate SiO_2_, a finding which shows the compensatory role for SiO_2_ of cellulose; lignin increased too, but its deposition in Si-deprived plants coincided with its localization in Si-supplemented rice (Yamamoto et al., 2012).

### Effects of Si on Plant Metabolism

The aspects linked to the effects of Si on plant metabolism are related to its priming role, which will be discussed in the next paragraph. The literature is constellated by many reports showing the effects of Si nutrition on plant metabolism and we will hereby discuss a few of the most recent results.

In miniature roses, Si was shown to induce an accumulation of fungitoxic phenolic compounds (chlorogenic acids and flavonoids) which correlated with ca. 50% reduction in disease severity when inoculation with *Podosphaera pannosa* was carried out (Shetty et al., 2011). This increase was accompanied by the enhanced expression of genes involved in the phenylpropanoid pathway (phenylalanine ammonia lyase, cinnamyl alcohol dehydrogenase, chalcone synthase). Interestingly, in this study, the supplementation of Si alone induced an increase in transcript abundance for these genes in the fifth developed leaves of miniature roses (Shetty et al., 2011). The production of flavonoids and organic acids can be a strategy adopted by plants to face exogenous stresses. These compound contribute for example to the chelation of heavy metals: catechin and quercetin can chelate Al, while malate and aconitate (whose synthesis is also stimulated by Si) can complex Cu (Adrees et al., 2015 and references therein).

Si-supplemented perennial ryegrass exposed to *Magnaporthe oryzae* was shown to accumulate chlorogenic acids and flavonoids and displayed an increased expression of peroxidase, polyphenol oxidase, penylalanine ammonia lyase, lipoxygenase (Rahman et al., 2015).

Si can also alter plant metabolism by acting on the endogenous levels of plant growth regulators. Si supplementation in rice under heavy metal stress ameliorates the toxic effects of Cd/Cu in the roots by reducing lipid peroxidation and fatty acid desaturation and by modulating the levels of plant growth regulators (Kim et al., 2014). In particular, the levels of abscisic acid increased at increasing stress periods, while those of jasmonic acid decreased; salicilic acid content, on the other hand, did not significantly change in the presence of Si.

Another evidence for an effect of Si on plant (secondary) metabolism comes from a recent study on the medicinal plant *Lonicera japonica* (honeysuckle) under salt stress: this study showed that Si induces the accumulation of chlorogenic acids in honeysuckle and stimulates the activities of catalase and superoxide dismutase (Gengmao et al., 2015).

### Silicon and Priming: How is it Achieved?

It is well known that Si exerts a protective role in plants, however, the detailed mechanisms of this process remain still obscure. The previous paragraph has illustrated the action of Si on plant metabolism and the promotion of the synthesis of compounds that are toxic, e.g., in case of fungal infection. Si stimulates the defense response of plants and while it was at first assumed that it exerted a purely passive role, now evidence is accumulating that Si has a more complex mechanism of action. Si triggers induced resistance in plants and at the beginning it was proposed that the role was that of a mechanical “barrier” hindering fungal penetration; however, the passive “barrier” role does not explain the activation of defense response (i.e., the priming action) observed for instance in the *Arabidopsis*-powdery mildew pathosystem (Fauteux et al., 2006). One can indeed wonder the following: if the role of Si is purely mechanical, why would the plant activate its defense arsenal? The role of Si might hence not be solely due to a mechanical function. It is important to highlight also that the supplementation of Si alone to *Arabidopsis* resulted in expression changes of only two genes (Fauteux et al., 2006), a finding which supports the stress-activated effects of Si. The role of Si on plant metabolism seems therefore to be indirect. It should, however, be noted that a study on another model system, rice, showed that in unstressed plants 35 and 121 TFs were up- and downregulated, respectively, (Van Bockhaven et al., 2012).

It was proposed that Si might bind to the hydroxyl groups of proteins involved in cellular signaling, act as a second messenger, and/or sequester cations necessary for the activity of enzymes related to pathogen processes (Fauteux et al., 2005).

A very interesting model explaining the mode of action of Si has been recently proposed by Vivancos et al. (2015): by using *Arabidopsis* mutants deficient in salicylic acid-dependent response and expressing the wheat Si transporter (TaLsi1), the authors showed that, upon inoculation with the fungus *Golovinomyces cichoracearum*, the plants supplemented with Si were much more resistant than control plants and the transformed ones deprived of Si. The authors suggest that the mode of action of Si takes place at the interface between plasma membrane and apoplast (a site where amorphous Si is actually deposited) and implies an interference with effectors. This means that Si prevents effectors from reaching their targets (Vivancos et al., 2015).

This is a very interesting perspective highlighting a cell wall-related mode of action of Si.

An apoplast-linked mode of action of Si was also hypothesized by Ye et al. (2013): in rice, the deposition of SiO_2_ might trigger a mild cell wall stress by interacting with cell wall components and this might be linked to the increase in jasmonic acid observed in rice plants infested with insects.

By taking into account the site of action of Si at the interface between cell wall-plasma membrane and the potential stress that the polymerization of Si might exert on the cell wall, it is tempting to speculate that the priming mechanism is (partly) due to the activation of the cell wall integrity maintenance (Hamann, 2015). This hypothesis would unify the observations by Ye et al. (2013) and Vivancos et al. (2015): wall-associated kinases (WAKs) are strategically located at the interface between cell wall and plasma membrane (exactly the site where amorphous Si is deposited), hence they might be involved in the response to eventual cell wall-localized stresses induced upon interaction of Si with cell wall components. Given the availability of a broad collection of *Arabidopsis* mutants, in the future this hypothesis could be tested by designing an experiment where WAKs mutants grown in the presence/absence of Si are exposed to a biotic stress and their responses are analyzed via, e.g., transcriptomics.

## Author Contributions

All authors listed, have made substantial, direct and intellectual contribution to the work, and approved it for publication.

## Conflict of Interest Statement

The authors declare that the research was conducted in the absence of any commercial or financial relationships that could be construed as a potential conflict of interest.
